# Maternal health outcomes in the context of fragility: a retrospective study from Lebanon

**DOI:** 10.1186/s13031-023-00558-1

**Published:** 2023-12-13

**Authors:** Hani Dimassi, Mohamad Alameddine, Nadine Sabra, Nour El Arnaout, Ranime Harb, Randa Hamadeh, Faysal El Kak, Abed Shanaa, Marta Orozco Mossi, Shadi Saleh, Natally AlArab

**Affiliations:** 1 School of Pharmacy, Lebanese American University, Beirut, Lebanon; 2 College of Health Sciences, University of Sharjah, Sharjah, United Arab Emirates; 3 Global Health Institute, American University of Beirut, Beirut, Lebanon; 4 Ministry of Public Health (MOPH), Beirut, Lebanon; 5 Faculty of Health Sciences, American University of Beirut (AUB), Beirut, Lebanon; 6 Department of Obstetrics Gynecology, American University of Beirut, Medical Center (AUB) Medical Center, Beirut, Lebanon; 7 United Nations Relief and Works Agency for Palestine Refugees in the Near East (UNRWA), Beirut, Lebanon; 8 Aflatoun International, Valencian Community, Spain; 9 Department of Health Management and Policy, Faculty of Health Sciences, American University of Beirut, Beirut, Lebanon

**Keywords:** Maternal health, Antenatal care, Primary healthcare, Fragility, Refugees, Disadvantaged pregnant women, Lebanon

## Abstract

**Background and aims:**

The Lebanese healthcare system faces multiple challenges including limited capacities, shortage of skilled professionals, and inadequate supplies, in addition to hosting a significant number of refugees. While subsidized services are available for pregnant women, representing the majority of the refugee population in Lebanon, suboptimal access to antenatal care (ANC) and increased maternal mortality rates are still observed, especially among socioeconomically disadvantaged populations. This study aimed to review the maternal health outcomes of disadvantaged Lebanese and refugee pregnant women seeking ANC services at primary healthcare centers (PHCs) in Lebanon.

**Methods:**

A retrospective chart review was conducted at twenty PHCs in Lebanon, including Ministry of Public Health (MOPH) and United Nations Relief and Works Agency for Palestine refugees (UNRWA) facilities. Data was collected from medical charts of pregnant women who visited the centers between August 2018 and August 2020. Statistical analysis was performed to explore outcomes such as the number of ANC visits, delivery type, and onset of delivery, using bivariate and multivariable logistic regression models.

**Results:**

In the study, 3977 medical charts were analyzed. A multivariate logistic regression analysis, revealed that suboptimal ANC visits were more common in the Beqaa region and among women with current abortion or C-section. Syrians had reduced odds of C-sections, and Beqaa, Mount Lebanon, and South Lebanon regions had reduced odds of abortion. Suboptimal ANC visits and history of C-section increased the odds of C-section and abortion in the current pregnancy. As for preterm onset, the study showed an increased likelihood for it to occur when being Palestinian, having current C-section delivery, experiencing previous preterm onset, and enduring complications at the time of delivery.

**Conclusion:**

This study suggests the need for low-cost interventions aiming at enhancing access to ANC services, especially among pregnant women in fragile settings.

## Background

Every woman has the right to safe and respectful maternal healthcare. Human rights standards surrounding safe pregnancy, childbirth, and respectful maternal care are rooted in the human rights to life, health, equality, and non-discrimination [1]. Governments must ensure these rights by creating and enabling conditions that support healthy women, pregnancies, and births [1]. Ensuring healthy lives and promoting well-being are being targeted by the United Nations’ (UN) Sustainable Development Goals (SDGs), specifically in SDG3 [2]. Two key targets under this goal by 2030 include reducing the global maternal mortality ratio to less than 70 per 100,000 live births and ensuring universal access to sexual and reproductive health-care services, including family planning, information and education, and integration of reproductive health into national strategies and programs [2]. Safeguarding maternal health and ensuring good maternal health outcomes is a great challenge in the context of fragility [3–5]. In Lebanon, an example of complex fragility context, assessing the local maternal health outcomes is imperative [6].

### Overview of the Lebanese healthcare system

The Lebanese healthcare system has long been characterized by its intricacy due to the engagement of many players in the health sector including the government, public and private healthcare service providers, non-governmental organizations (NGOs), third party payers, among others [7]. There are several factors overburdening the healthcare system in Lebanon, one of which is the significant number of refugees fleeing conflicts from neighboring countries and residing in mostly the underserved and marginalized areas of Lebanon [8]. In fact, Lebanon hosts the highest number of refugees per capita globally, with approximately 1.5 million Syrian refugees, 452,669 Palestinian refugees, and 12,159 refugees of other nationalities residing in the country [9, 10]. When the system faces a combination of risks and lacks adequate resources to effectively mitigate them, it is often referred as fragility [11].

With refugees settling in underserved areas, they further stretch the already limited and depleted resources of host communities which further compounds the fragility of care systems for both refugees and local hosts [12]. Therefore, fragility renders populations susceptible to a spectrum of challenges, encompassing issues such as poverty, insecurity, and disparities in social and economic well-being [13]. Consequently, the multi-layered crisis that the country has been witnessing since 2019 including political instability, a deteriorating economy, and the COVID-19 pandemic aggravated the burden on the Lebanese healthcare system [14]. This strain was already evident due to limited capacities, shortage in skilled health professionals, as well as limited supply of medications and equipment [14–16]. Additionally, it has been revealed that women's health, among other critical healthcare concerns, represents a central concern for refugees in Lebanon, further underscoring the complexities faced by the healthcare system [17].

### Primary healthcare service utilization among disadvantaged populations in Lebanon

Disadvantaged Lebanese individuals and Syrian refugees often seek primary health services at primary healthcare centers (PHCs) through the National PHC Network established by the Lebanese Ministry of Public Health (MOPH), which includes around 258 PHCs across Lebanon, mostly operated by NGOs, municipalities, and governmental institutions [18, 19]. The Network’s PHCs annually serve more than 1 million Syrian refugees and disadvantaged Lebanese individuals, through a variety of low-cost primary healthcare services (e.g. medical consultations, diagnostics (laboratory tests and imaging), etc.) including reproductive and maternal health services and pediatric services, among others [20, 21]. While Lebanese individuals pay discounted fees out of pocket at the PHCs, Syrian refugees receive the PHCs’ services with limited copay through the support of subsidies from the United Nations High Commissioner for Refugees (UNHCR) [18, 22]. On the other hand, most Palestinian refugees access healthcare services across Lebanon through a network of 27 PHCs governed by the United Nations Relief And Works Agency for Palestine Refugees in the Near East (UNRWA) [10]. Similar to MOPH’s PHCs, UNRWA’s PHCs offer a wide range of PHCs’ services including general consultations [10]. Among all the rights to which humans are entitled, healthcare is unquestionably one of the most intersectional and fundamental ones. Assuring health coverage in underprivileged communities is crucial to the ability of marginalized segments of any population to live lives of dignity. Universal Health coverage has also been termed a “practical expression of the right to health” [23].

### Status of maternal health among disadvantaged populations in Lebanon

Women of childbearing age represent the majority of the refugee population, and constitute the highest proportion of beneficiaries visiting the PHCs across Lebanon [24, 25]. In fact, UNHCR subsidizes a wide range of antenatal and maternal health services targeting registered pregnant Syrian women [22]. This includes 75% coverage for delivery costs, 85% coverage for laboratory tests, full coverage for two ultrasounds, and most of the fee for four recommended antenatal care (ANC) visits [22]. Other subsidized services include family planning services, breastfeeding awareness sessions, and postnatal care (PNC) [22]. Similarly, access to ANC, PNC, delivery care, and family planning services is also supported by the UNRWA for the Palestinian refugees [26].

While women’s health services represent the most common health need among refugees in Lebanon [27], suboptimal access to ANC visits is still evident due to various factors including socioeconomic factors, proximity of health centers to the residing area of the pregnant women and associated transportation cost, among others [28–31]. Despite the effort invested by multiple stakeholders to enhance access to primary ANC services, increased maternal mortality rates and pregnancy-related complications such as anemia, hemorrhage, urinary tract infections, gestational diabetes and others were still recorded, mostly among socioeconomically disadvantaged populations [29, 32, 33]. Literature suggests that the increase in maternal health complications, particularly those requiring intensive care unit admissions and blood transfusions, could be related to the cesarean delivery [34, 35].

While there are several studies reflecting the state of maternal health in Lebanon [32, 36–39], very few focus on recent health data of disadvantaged women, particularly in primary healthcare settings [40, 41].This gap in the availability of recent data may be attributed to several factors including the migrating nature of disadvantaged populations [42], the lack of systematic data collection and follow-up, and the COVID-19 pandemic which significantly shifted the priority agenda for many researchers. In addition to that, many studies focus on testing specific interventions that aim to improve maternal heath [43, 44], skipping as such the important examination of baseline data and maternal health status of the targeted population.

### Study aim

Given the scarcity of recent data on health outcomes of pregnant women in Lebanon, especially among disadvantaged populations, this study aims to review the maternal health outcomes of disadvantaged Lebanese and refugee pregnant women seeking ANC services at PHCs in Lebanon. This study will therefore provide a baseline to assess any future improvement in maternal health outcomes that may result from implemented interventions in this field.

## Methods

### Study design

**Fig. 1:**
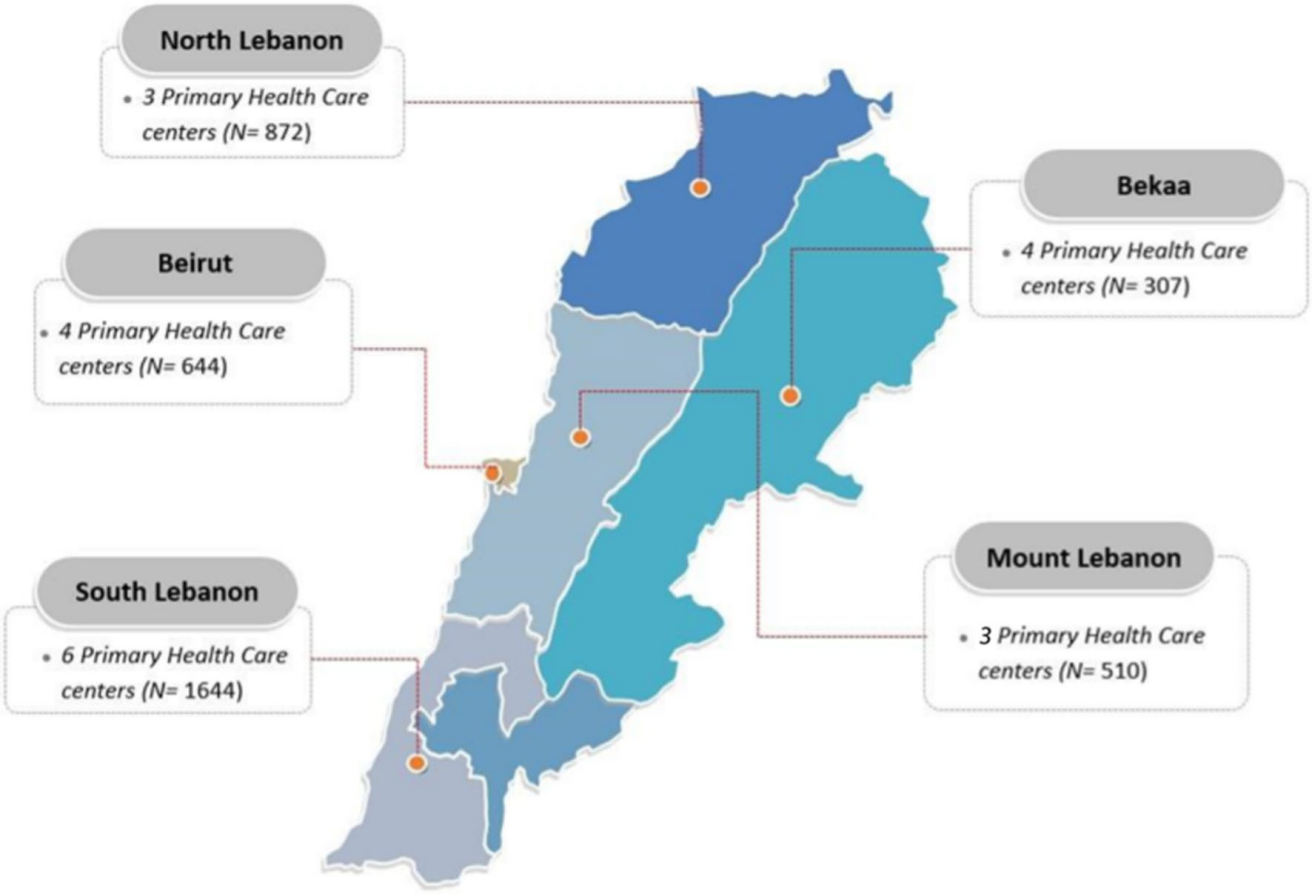
The geographic distribution of 20 selected Primary Healthcare centers (PHCs) affiliated with the Ministry of Public Health (MOPH) and United Nations Relief and Works Agency (UNRWA) providing maternal Health services for disadvantaged women and refugees in Lebanon (N = 3977). *N* represents the number of pregnant women in that have been enrolled in the analysis

A retrospective chart review was conducted at twenty PHCs that provide ANC services to disadvantaged Lebanese and refugee (namely Syrian and Palestinian) women in five governorates of Lebanon, including Beirut, Bekaa, North Lebanon, Mount Lebanon, and South Lebanon ***(***Fig. 1***)***. Out of these twenty PHCs, ten were part of the National PHC Network established by the MOPH and provided ANC services to disadvantaged Lebanese and Syrian refugee women. The other ten PHCs were run by UNRWA and provided ANC services to Palestinian refugee women. At least one MOPH PHC and one UNRWA PHC were selected from each governorate. Ethical approval was sought and obtained from the Institutional Review Board at the American University of Beirut and the respective ethical committees at MOPH and UNRWA.

### Data collection

Data collection was carried out between July and December 2021. Data was collected from the medical charts of pregnant women who were Lebanese nationals, Syrian refugees and Palestinian refugees. Pregnant women were included in the study if they visited one of the PHCs included in this study for ANC on their first trimester (up to 12 weeks of gestation) between August 2018 and August 2020, and continued visiting the center until their delivery or termination of the pregnancy. In case where a woman had multiple pregnancies during the indicated period of interest, only data from the latest pregnancy that met the eligibility criteria was considered. Data collection was performed by staff from the PHCs including nurses and midwives, trained by the research team. Data was abstracted in English or Arabic. The collected variables included (1) demographics such as nationality, area of residency, and age of the pregnant women, (2) medical history (e.g. chronic diseases, etc.) and surgical history (excluding c-sections and Dilation and curettage), (3) obstetric history such as number of gravidae, parities, preterm deliveries, abortions and c-sections, (4) antenatal history of current pregnancy of interest such as gestational age at first visit, number of completed ANC visits, labs and ultrasound imaging done during the pregnancy of interest, vaccines taken, and pregnancy-related complications (5) outcomes of the current pregnancy of interest including delivery onset and method, and complications during delivery, and (6) postnatal history of current pregnancy of interest such as postnatal complications, neonatal complications, number of PNC visits completed and breastfeeding practices.

### Statistical analysis

The data collected from the medical charts was coded into an Excel sheet and exported for analysis using SPSS V27. Categorical data was presented using frequency and percent. Numerical data was presented using mean and standard deviations. Three variables were considered as outcomes of interest: the number of ANC visits (categorized into less than 4 visits and 4 or more visits based on WHO recommendation on number of visits [45]), type of delivery/delivery outcome (normal, c-section, or abortion), and onset of delivery (term and preterm). Bivariate analysis was used to explore the percentage of suboptimal outcomes including < 4 ANC visits, c-section, abortion, and preterm onset of delivery vis-à-vis each of the independent variables. Independent variables were grouped by women characteristics, supplement/lab tests/ultrasounds, and pregnancy history. Differences in proportions were tested using Pearson chi-square except for gravida and parity where ANOVA f test was used after checking normal distribution of both variables. Three multivariable logistic regression models were built, one per outcome variable, using the stepwise method and including all independent variables with p < 0.20 at bivariate analysis in the initial model. Analyses were carried out at a significance level of 0.05.

## Results

A total of 3977 medical charts were included in the analysis (Table 1). Approximately 67% of pregnant women attending the PHC centers were Palestinians, 22% were Syrian, and 10.4% were Lebanese. There were also a small number (n = 24) of women from various other nationalities, Bengali (n = 7), Ethiopian (n = 1), Iraqi (n = 8), Jordanian (n = 6), Moroccan (n = 1), Russian (n = 1). However, due to the limited representation of these nationalities, they were excluded from the analysis. Most pregnant women were within the 18 to 39 years age group (94%). Only 5% had a documented history of chronic disease, with obesity and cardiovascular diseases being the most common. About 6.2% of these women reported previous non-obstetric surgical history. Most women had previous pregnancies with a mean gravida of 3 and a mean parity of 1.6. Around 29% of women had documented C- section delivery, 6.3% reported preterm deliveries, and 29.3% reported abortion cases.

**Table 1:** Socio-demographics, obstetric history, and pregnancy outcomes of pregnant women receiving maternal health services at selected PHC centers in Lebanon

	N	%
Demographics		
Nationality		
Lebanese	412	10.4%
Syrian	875	22.0%
Palestinian	2690	67.6%
Age Of Pregnant Women		
≤ 17	94	2.4%
18–28	2137	53.7%
29–39	1601	40.3%
≥ 40	145	3.6%
Previous Medical History		
Chronic Disease	209	5.3%
Obesity	71	1.8%
Cardiovascular Diseases	60	1.5%
Hypertension	48	1.2%
Diabetes Mellitus	27	0.7%
Obstetric History of previous pregnancies		
Mean Gravidity (SD)	3.08	(1.857)
Mean Parity (SD)	1.63	(1.466)
Preterm Delivery (yes)	249	6.3%
Abortion (Yes)	1178	29.6%
C-Section (yes)	1139	28.8%
Obstetric History of last pregnancy		
Antenatal care (ANC) visits		
< 4	1207	30.3%
≥ 4	2770	69.7%
Ultrasound of Uterus		
None	589	14.8%
1	1626	40.9%
≥ 2	1762	44.3%
Labs Done		
CBC (yes)	3602	90.6%
Fasting Blood Sugar (yes)	3432	86.3%
Oral Glucose Tolerance Test (yes)	1042	26.2%
Ferritin Test (yes)	187	4.7%
Urinalysis Test (yes)	3073	77.3%
Hepatitis B (Hbsag)*	757	83.8%
Rubella Titer (Igg)* (yes)	823	91.6%
Toxoplasma Titer (Igg)* (yes)	498	90.3%
Syphilis (VDRL)*(yes)	767	83.6%
HIV Test* (yes)	829	91.7%
Blood Group/Rhesus Factor (yes)	3594	90.4%
Supplements Taken at last pregnancy		
Folic Acid	3744	94.5%
Iron	3473	87.6%
Received Tetanus-diphtheria vaccine* (yes)	2511	63.2%
Last pregnancy outcome		
Normal	2073	52.1%
C-Section	1590	40.0%
Abortion	314	7.9%
Delivery Onset		
Preterm	456	13.3%
Term	2932	85.6%
Post Term	37	1.1%
Obstetric Complications of last pregnancy		
During pregnancy complications (yes)		
Gestational Hypertension	66	1.7%
Gestational Diabetes	84	2.2%
Perinatal Depression	59	1.6%
At delivery complications (yes)		
Still Birth	20	0.5%
Postpartum Complications (yes)		
Infection	11	0.3%
Hemorrhage	19	0.5%
Depression	32	0.8%
Neonatal Complications (yes)		
NICU Admission	59	1.6%
Spontaneous Preterm	77	2.1%
Birth Injury	5	0.1%
Low Birth Weight	106	2.7%
Early Neonatal Death	47	1.3%
Postnatal History		
Postnatal care (PNC) visits (yes)	3345	84.1%
Breastfeeding (yes)	2837	85.2%

*Lab tests and vaccines not provided at all PHC centers included in this study

### Obstetric history and outcomes of latest pregnancy among pregnant women

Out of the total sample of included pregnant women, 69.7% attended PHCs at least 4 times for ANC during their most recent pregnancy (pregnancy of interest in this study). Uterine ultrasound imaging was done more than two times per pregnancy among 44.3% of the included pregnant women with charts indicating that only 14.8% of women haven’t undergone any sonography during their pregnancy. The majority of pregnant women had received oral supplements and underwent the necessary pregnancy lab workout including CBC, blood sugar tests and urine analysis, blood grouping, in addition to blood serology for hepatitis B, rubella, toxoplasma, syphilis and HIV. Ferritin test on the other hand was reported in only 4.7% of the sample. Tetanus-diphtheria vaccine was given to 63% of the women.

Reviewed medical charts showed that 92.1% of the pregnant women gave birth successfully, with 40% undergoing C-section delivery. Term deliveries represented the majority (85.6%). Abortion cases accounted for 7.9% of pregnancies representing either elective or spontaneous abortion. Complications during pregnancy were rarely documented with gestational diabetes (2.2%) gestational hypertension (1.7%) and perinatal depression (1.6%) being the most common. Stillbirth and postpartum complications were very rarely reported. The incidence rate for stillbirth was 0.5% and postpartum complications were portrayed as follows 0.3%, 0.5%, and 0.8% representing infection, hemorrhage, and depression, respectively. Similarly, neonatal complications were low, with low birth weight and preterm delivery onset not exceeding 2.7%. Almost 84.1% of the pregnant women had at least one PNC visit and 85% reported breastfeeding (Table 1).

### Characteristics of pregnant women attending PHC centers by ANC visits, type of delivery/delivery outcome, and delivery onset

Table 2 highlights the associations of the three dependent variables-outcomes: ANC visit, type of delivery/delivery outcome, and delivery onset. All three outcomes were significantly associated with the PHC region (*p*-values < 0.001 each). The Bekaa region exhibited the highest rates of low ANC visits (72.3%) and preterm onset (33.1%). Beirut had the highest rates of c-Sect. (44.1%), which is comparable to that of pregnant women in Bekaa (42.3%) and South of Lebanon (43.0%). Beirut also had the highest rate for abortion (12.4%). Nationality was associated with both number of ANC visits and type of delivery/delivery outcome (*p*-values < 0.001), but no difference was observed with preterm onset (*p*-value = 0.216). Lebanese and Syrian women had low ANC visits (37%) compared to Palestinians (26.8%). The highest rate of c-section was among Palestinians (47.1%), followed by Lebanese (38.6%), and Syrians (18.7%). Abortion rates were highest among Palestinians (8.5%) and Lebanese (9%), compared to those of Syrians women (5.5%).

**Table 2:** Characteristics of pregnant women attending primary healthcare centers by antenatal care (ANC) visits, delivery outcome, and delivery onset of latest/most recent pregnancy

	Number of ANC visits			Delivery outcome					Delivery onset		
	ANC < 4			C-section		Abortion			Preterm		
	N	%	*p*-value	N	%	N	%	p-value	N	%	*p*-value
*PHC Region*			** < .001**					** < .001**			** < .001**
Beirut	180^ab^	28.0%		284^a^	44.1%	80^a^	12.4%		64^a^	13.1%	
Mount Lebanon	133^bd^	26.1%		173^b^	33.9%	33^d^	6.5%		56^a^	11.9%	
Beqaa	222^c^	72.3%		130^a^	42.3%	24^bcd^	7.8%		78^b^	33.1%	
South Lebanon	391^d^	23.8%		707^a^	43.0%	91^ac^	5.5%		183^a^	12.2%	
North Lebanon	281^a^	32.2%		296^b^	33.9%	86^bd^	9.9%		75^a^	10.8%	
*Nationality*			** < .001**					** < .001**			.216
Lebanese	154^a^	37.4%		159^a^	38.6%	37^a^	9.0%		49	14.7%	
Syrian	331^a^	37.8%		164^b^	18.7%	48^b^	5.5%		95	15.3%	
Palestinian	722^b^	26.8%		1267^c^	47.1%	229^a^	8.5%		312	12.8%	
*Age groups*			**.003**					** < .001**			**.003**
< = 17	28^a^	29.8%		22^a^	23.4%	5^ab^	5.3%		4^a^	5.4%	
18–28	627^a^	29.3%		798^b^	37.3%	138^b^	6.5%		221^ab^	12.0%	
29–39	488^a^	30.5%		727^c^	45.4%	133^a^	8.3%		216^c^	15.8%	
40 +	64^b^	44.1%		43^ab^	29.7%	38^c^	26.2%		15^bc^	15.0%	
Delta Gravida: Mean (SD)*	0.43	(0.07)	** < .001**	-0.03	(0.06)	0.54	(0.12)	** < .001**	0.50	(0.10)	** < .001**
Delta Parity: Mean (SD)*	0.29	(0.05)	** < .001**	-0.12	(0.05)	0.21	(0.09)	**.001**	0.29	(0.08)	** < .001**
History of Abortion			** < .001**					** < .001**			** < .001**
Yes	415	35.2%		504^a^	42.8%	130^a^	11.0%		169	17.5%	
No	792	28.3%		1086^b^	38.8%	184^b^	6.6%		287	11.9%	
*History of C-section*			.460					** < .001**			** < .001**
Yes	355	31.2%		980^a^	86.0%	101^a^	8.9%		191	19.2%	
No	845	30.0%		604^b^	21.4%	212^a^	7.5%		263	11.1%	
*History of Preterm delivery*			**.006**					** < .001**			** < .001**
Yes	95	38.2%		132^a^	53.0%	15^a^	6.0%		70	34.3%	
No	1112	29.8%		1458^b^	39.1%	299^a^	8.0%		386	12.1%	
*History of Chronic Disease*			**.029**					**.006**			.431
Yes	49	23.4%		104^a^	49.8%	18^a^	8.6%		28	15.4%	
No	1143	30.6%		1470^b^	39.3%	294^a^	7.9%		424	13.3%	

Bold means ‘statistically significant p-value’

When more than 2 groups are compared, superscript letters are used to depict statistical differences

*Delta values in table represent differences between group shown and reference group not shown (example ANC < 4 – ANC >  = 4)

ANC visits was the lowest among women aged above 40 (44.1%, *p*-value = 0.003). C-section was most reported among those aged 29–39 (45.4%), while abortion rates were highest among women above the age of 40 (26.2%) (*p*-value < 0.001). Preterm onset was as low as 5% among very young mothers (age 17 or younger), while the rate was above 12% among other age groups (*p*-value = 0.003). On the other hand, women with < 4 ANC visits reported on average more gravidae and parities than their counter parts (mean difference of 0.4 and 0.3 respectively, p-value < 0.001). Women whom charts reported documented abortion during the current pregnancy had more gravidae (on average 0.54 higher mean *p* < 0.001), and higher parity (overage 0.2 higher), while women with documented C-sections reported higher parity as well (0.12 higher than normal women with normal delivery outcome, *p* < 0.001). Similar results were observed in women with preterm delivery, with results revealing higher gravidae and parity (mean difference of 0.5 and 0.3; respectively (*p* < 0.001)). Women with history of abortion were more likely to have lower ANC visits (35.2% vs. 28.3% *p* < 0.001) and higher rates of c-Sect. (42.8% vs. 38.8%), abortion (11% vs 6.6%) (*p* < 0.001), and pre-term delivery (17.5% vs 11.9%, *p*-value < 0.001). Similar trends were observed in women with previous c-section, and preterm deliveries.

### Supplements use and diagnostics by ANC visits, type of delivery/delivery outcome, and delivery onset

Associations between supplements and lab tests with the three outcomes are presented in Table 3. In general, women who did not receive supplements or did not perform necessary lab test were more likely to exhibit negative outcomes including < 4 ANC visits, c-section, abortion, and preterm delivery. For instance, pregnant women who reported receiving iron supplement were observed to have lower rates of abortion (2.8% vs 43.5% *p* < 0.001). However, some exceptions to this trend were observed with women who reported doing FBS and Hepatitis B test having higher rates of abortion (41.1% vs 32.8% *p* < 0.001, and 16.1% vs 11.0% *p* < 0.001, respectively). In terms of imaging, pregnant women who did not undergo any ultrasounds had higher rates of low ANC visits (< 4), c-section, and abortion.

**Table 3:** Supplements use, laboratory tests, and ultrasound imaging for pregnant women attending PHC centers by ANC visits, delivery outcome and delivery onset of latest/most recent pregnancy

	Number of ANC visits			Delivery outcome					Delivery onset		
	ANC < 4			C-section		Abortion			Preterm		
	N	%	p-value	N	%	N	%	*p*-value	N	%	*p*-value
*Supplements*											
Folic Acid (yes)	1129	30.2%	.996	1540^a^	41.1%	297^a^	7.9%	** < .001**	426	13.2%	.054
Folic Acid (no)	66	30.1%		48^b^	21.9%	13^a^	5.9%		30	18.5%	
Iron (yes)	859	24.7%	** < .001**	1505^a^	43.3%	97^a^	2.8%	** < .001**	417	13.0%	**.001**
Iron (no)	336	68.6%		83^b^	16.9%	21^b^	43.5%		39	21.5%	
*Lab Tests*											
CBC (yes)	1065	29.6%	**.001**	1433^a^	39.8%	273^a^	7.6%	.028	403	13.1%	.052
CBC (no)	142	37.9%		157^a^	41.9%	41^b^	10.9%		53	17.0%	
FBS (yes)	965	28.1%	** < .001**	1411^a^	41.1%	245^a^	7.1%	** < .001**	391	13.2%	.301
FBS (no)	242	44.4%		179^b^	32.8%	69^b^	12.7%		65	15.0%	
OGTT (yes)	158	15.2%	** < .001**	350^a^	33.6%	31^a^	3.0%	** < .001**	87	9.6%	** < .001**
OGTT (no)	338	46.9%		293^b^	40.6%	101^b^	14.0%		92	15.8%	
Ferritin (yes)	69	36.9%	**.046**	25^a^	13.4%	7^a^	3.7%	** < .001**	1	0.8%	** < .001**
Ferritin (no)	1138	30.0%		1565^b^	41.3%	307^b^	8.1%		455	14.0%	
Urinalysis (yes)	888	28.9%	** < .001**	1197^a^	39.0%	226^a^	7.4%	**.001**	334	12.8%	.047
Urinalysis (no)	319	35.3%		393^b^	43.5%	88^b^	9.7%		122	15.6%	
Hepatitis B (yes)	251	33.2%	** < .001**	122^a^	16.1%	27^a^	3.6%	** < .001**	99	17.2%	.077
Hepatitis B (no)	104	71.2%		16^a^	11.0%	25^b^	17.1%		2	5.7%	
Rubella (yes)	299	36.3%	** < .001**	123^a^	14.9%	32^a^	3.9%	** < .001**	98	16.5%	.626
Rubella (no)	56	74.7%		15^a^	20.0%	20^b^	26.7%		3	21.4%	
Syphilis (yes)	253	33.0%	** < .001**	134^a^	17.5%	28^a^	3.7%	** < .001**	102	17.3%	.066
Syphilis (no)	107	70.9%		18^a^	11.9%	26^b^	17.2%		2	5.6%	
HIV (yes)	299	36.1%	** < .001**	125^a^	15.1%	32^a^	3.9%	** < .001**	97	16.2%	.413
HIV (no)	58	77.3%		17^b^	22.7%	21^b^	28.0%		3	25.0%	
Toxoplasmosis (yes)	292	36.6%	** < .001**	125^a^	15.7%	32^a^	4.0%	** < .001**	103	18.2%	**.001**
Toxoplasmosis (no)	61	70.9%		16^a^	18.6%	19^b^	22.1%		2	6.5%	
*Ultrasounds done*			** < .001**					** < .001**			** < .001**
None	311^a^	52.8%		253^a^	43.0%	105^a^	17.8%		56^a^	12.0%	
1	496^a^	30.5%		674^a^	41.5%	187^b^	11.5%		225^b^	16.9%	
≥ 2	400^c^	22.7%		663^b^	37.6%	22^c^	1.2%		175^a^	11.0%	

Bold means ‘statistically significant p-value’

### *Pregnancy history and pregnancy-related complications among pregnant women attending PHCs by ANC visits, type of delivery/delivery outcome and delivery onset* of latest/most recent pregnancy

The association of the three outcomes of interest with each other, in addition to pregnancy-related complications is presented in Table 4. Completing less than the recommended number of ANC visit (< 4) was associated with higher rates of abortion (25% vs. 0.3% *p* < 0.001) and preterm delivery (18% vs. 12.2% *p* < 0.001). Having neonatal adverse outcome was associated with higher rates of c-section and preterm delivery (*p*-values < 0.001), while no association was observed between neonatal adverse outcomes and the number of ANC visits (*p* = 0.815). Interestingly, women who reported having pregnancy-related complications (e.g. gestational diabetes) were less likely to have completed the recommended number of ANC visits (11.9% vs 28.9% *p* < 0.001). A similar trend was observed with women reporting postpartum adversities (infection, hemorrhage, or depression) (11.3% vs 28.3% *p* = 0.003).

**Table 4:** Pregnancy history, delivery outcome, and pregnancy complications of pregnant women attending PHC centers by ANC visits, delivery outcome and delivery onset of latest/most recent pregnancy

	Number of ANC visits			Delivery outcome							Delivery onset		
	ANC < 4			C-section			Abortion				Preterm		
	N	%	p-value	N		%	N		%	p-value	N	%	p-value
*ANC visits*										** < .001**			** < .001**
< 4	–	–	–	399^a^		33.1%	307^a^		25.4%		133	18.0%	
≥ 4	–	–	**–**	1191^b^		43.0%	7^b^		0.3%		323	12.2%	
*Delivery outcome*			** < .001**										** < .001**
Normal	501^a^	24.2%		–		–	–		–		162	8.7%	
C-section	399^a^	25.1%		–		–	–		–		294	19.2%	
Abortion	307^b^	97.8%		–		–	–		–		N/A	N/A	
*Delivery onset*			** < .001**							** < .001**			
Preterm	133^a^	29.2%		294^a^		64.5%	–		–		–	–	–
Term	604^b^	20.6%		1239^b^		42.3%	–		–		–	–	–
Neonate Adverse outcomes (yes)	61	28.5%	.815	135^a^		63.1%	9^a^		4.2%	** < .001**	121	60.5%	** < .001**
Neonate Adverse outcomes (no)	981	27.8%		1391^b^		39.4%	275^a^		7.8%		287	9.3%	
NICU Admission (yes)	12	20.3%	.197	50^a^		84.7%	–^a^		–	** < .001**	35	59.3%	** < .001**
NICU Admission (no)	1030	27.9%		1476^b^		40.0%	284^b^		7.7%		373	11.6%	
Spontaneous Preterm (yes)	19	24.7%	.535	48^a^		62.3%	2^a^		2.6%	** < .001**	63	85.1%	** < .001**
Spontaneous Preterm (no)	1023	27.9%		1478^b^		40.3%	282^b^		7.7%		345	10.8%	
Birth Injury (yes)	1	20.0%	.697	2		40.0%	–		–	.885	1	20.0%	.610
Birth Injury (no)	1041	27.8%		1524		40.7%	284		7.6%		407	12.5%	
Low Birth Weight (yes)	24	22.6%	.228	70^a^		66.0%	–^a^		–	** < .001**	65	61.9%	** < .001**
Low Birth Weight (no)	1018	28.0%		1456^b^		40.0%	284^b^		7.8%		343	10.8%	
Early Neonatal Death (yes)	22	46.8%	**.003**	24^a^		51.1%	7^a^		14.9%	**.024**	27	73.0%	** < .001**
Early Neonatal Death (no)	1020	27.6%		1502^a^		40.6%	277^a^		7.5%		381	11.8%	
*Adverse pregnancy outcomes*													
During Pregnancy (yes)	23	11.9%	** < .001**	71		36.8%	9		4.7%	.068	20	11.5%	.716
During Pregnancy (no)	1036	28.9%		1460		40.7%	290		8.1%		385	12.4%	
Gestational Hypertension (yes)	6	9.1%	**.001**	36		54.5%	3		4.5%	**.058**	13	21.3%	**.032**
Gestational Hypertension (no)	1053	28.4%		1495		40.3%	296		8.0%		392	12.2%	
Gestational Diabetes (yes)	14	16.7%	**.019**	38		45.2%	5		6.0%	.604	7	9.2%	.396
Gestational Diabetes (no)	1045	28.3%		1493		40.4%	294		8.0%		398	12.5%	
Perinatal Depression (yes)	6	10.2%	**.002**	6^a^		10.2%	1^a^		1.7%	** < .001**	2	3.9%	.065
Perinatal Depression (no)	1053	28.3%		1525^b^		41.0%	298^a^		8.0%		403	12.5%	
*At Delivery*													
Still Birth (yes)	9	45.0%	.091	7		35.0%	1		5.0%	.723	12	75.0%	** < .001**
Still Birth (no)	1050	28.0%		1524		40.6%	298		7.9%		393	12.1%	
Postpartum (yes)	7	11.3%	.**003**	21^a^		33.9%	–^a^		–	**.016**	7	11.5%	.829
Postpartum (no)	1052	28.3%		1510^a^		40.7%	299^b^		8.1%		398	12.4%	
Postpartum Infection(yes)	2	18.2%	.466	3		27.3%	–		–	.314	3	30.0%	.090
Postpartum Infection (no)	1057	28.1%		1528		40.6%	299		7.9%		402	12.3%	
Postpartum Hemorrhage (yes)	3	15.8%	.233	12		63.2%	–		–	.094	4	21.1%	.250
Postpartum Hemorrhage (no)	1056	28.1%		1519		40.4%	299		8.0%		401	12.3%	
Postpartum Depression (yes)	2	6.3%	**.006**	6^a^		18.8%	–^a^		–	**.003**	0	0.0%	**.033**
Postpartum Depression (no)	1057	28.2%		1525^b^		40.7%	299^a^		8.0%		405	12.5%	

Bold means ‘statistically significant p-value’

### Multivariable logistic regression

When all factors were entered into a multivariable logistic regression model (Table 5), a suboptimal number of visits was observed mostly in the Beqaa region (OR = 6.86, 95%CI 4.59 to 10.26). Additionally, women who had delivered via C-section (OR = 1.41, 95%CI 1.11 to 1.78) in their current pregnancy of interest or have experienced abortion (OR 44.5, 95%CI 17.53 to 112.96) also showed suboptimal number of visits. On the other hand, complications during the latest pregnancy, undergoing one, two and more ultrasounds, and urinalysis test were all associated with a lower likelihood of suboptimal ANC visits (OR = 0.32, 0.36, 0.45, and 0.59 respectively). As for the type of delivery/delivery outcome, Syrians had reduced odds of delivering via c-section (OR = 0.49 95%CI 0.31 to 0.79). Being located at the North or South of Lebanon reduced the odds of c-section by about 50% as well. Additionally, being from the Beqaa, Mount Lebanon, or South region reduced the odds of abortion by 86% and 66%; respectively. A suboptimal number of ANC visits (< 4 ANC visits) increased the likelihood of c-section and abortion (OR 1.45 and 250 respectively, *p*-values < 0.001). A history of abortion and c-section were both positively associated with the occurrence of c-section in the current pregnancy of interest (OR = 1.8 and 90.9 respectively *p*-value < 0.001). A history of c-section increased the likelihood of abortion during the current pregnancy of interest by 25-fold (OR = 25.64 95% CI 15.6 to 41.67). Having done CBC/FBS lab tests were associated with a reduction in the odds of c-section and abortion (OR = 0.29 and 0.18 respectively *p*-value < 0.001). Preterm delivery was shown to be associated with Palestinian nationality (OR = 1.69 95%CI 1.01 to 2.82), c-section delivery during the current pregnancy (OR = 2.85 *p* < 0.001), having a history of preterm delivery (OR = 2.36), experiencing complications at the time of delivery and neonatal complications (OR = 32 and 4.85; respectively *p*-value < 0.001). Furthermore, women with records of 1 and 2 or more ultrasounds had higher odds of preterm delivery (OR 1.58 95%Ci 1.03 to 2.42 and OR 1.61 95% CI 1.01 to 2.58). In terms of regions, women from Mount Lebanon and South Lebanon exhibited lower odds of preterm delivery (OR = 0.43 and 0.64 respectively), as did women who had a CBC/FBS lab test during pregnancy (OR 0.446 *p* = 0.01).

**Table 5:** Multivariable logistic regressions of ANC visits (≥ 4 visits versus < 4 ref), delivery outcome (C-section versus normal and abortion versus normal), and delivery onset (preterm versus term)

	ANC visits				Delivery Outcome								Delivery Onset			
	< 4 visits vs. ≥ 4 visits				C-section vs. Normal				Abortion vs. Normal				Preterm vs. Term			
	OR	Lower Limit	Upper Limit	P-value	OR	Lower Limit	Upper Limit	P-value	OR	Lower Limit	Upper Limit	P-value	OR	Lower Limit	Upper Limit	P-value
*Nationality*																
Lebanese (ref)	1.0				1.0				1.0				1.0			
Syrian	0.91	0.62	1.35	0.66	0.49	0.31	0.79	0.003	0.66	0.27	1.64	0.37	1.25	0.66	2.35	0.49
Palestinian	0.80	0.58	1.1	0.17	1.31	0.92	1.85	0.13	1.73	0.96	3.09	0.06	1.69	1.01	2.82	0.05
*Region*																
Beirut (ref)	1.0				1.0				1.0				1.0			
Beqaa	6.86	4.59	10.26	< .001	0.86	0.53	1.38	0.52	0.14	0.07	0.29	< .001	1.38	0.768	2.49	0.28
Mount Lebanon	1.75	1.19	2.57	0.004	0.82	0.57	1.18	0.28	0.34	0.18	0.64	0.001	0.43	0.258	0.73	0.002
North Lebanon	2.19	1.57	3.07	< .001	0.50	0.36	0.71	< .001	1.25	0.68	2.29	0.476	0.72	0.447	1.14	0.16
South Lebanon	1.45	1.06	1.97	0.02	0.51	0.38	0.68	< .001	0.34	0.19	0.59	< .001	0.64	0.43	0.94	0.03
*Age groups*																
< = 17 (ref)	1.0				1.0			1.0					1.0			
18–28	1.33	0.71	2.48	0.37	0.80	0.43	1.46	0.47	0.44	0.11	1.67	0.23	2.39	0.65	8.81	0.19
29–39	1.19	0.63	2.27	0.59	1.06	0.57	1.97	0.86	0.68	0.17	2.69	0.59	2.53	0.68	9.43	0.17
40 +	0.85	0.36	1.97	0.69	1.16	0.49	2.77	0.73	3.46	0.73	16.32	0.12	1.13	0.24	5.43	0.88
*Gravida*																
Per unit increase	1.08	1.01	1.15	0.03	0.67	0.62	0.73	< .001	0.85	0.75	0.97	0.02	1.07	0.98	1.17	0.14
ANC Visits																
< 4 vs ≥ 4 (ref)	NA	NA	NA	NA	1.45	1.15	1.84	0.002	250.00	111.11	500.00	< .001	0.89	0.64	1.22	0.46
*Onset of Delivery*																
Term (ref)	1.0															
Preterm	0.91	0.67	1.24	0.55	NA	NA	NA	NA	NA	NA	NA	NA	NA	NA	NA	NA
Post-term	2.42	1.08	5.41	0.03	NA	NA	NA	NA	NA	NA	NA	NA	NA	NA	NA	NA
*Delivery Method*																
Normal (ref)	1.0												1.0			
C-section	1.41	1.11	1.78	0.01	NA	NA	NA	NA	NA	NA	NA	NA	2.85	2.06	3.94	< .001
Abortion	44.50	17.53	112.96	< .001	NA	NA	NA	NA	NA	NA	NA	NA	NA	NA	NA	NA
*History of*																
Abortion	1.07	0.85	1.34	0.56	1.80	1.39	2.33	< .001	1.51	0.98	2.35	0.07	1.09	0.79	1.5	0.58
C-Section	0.79	0.62	1.03	0.08	90.91	66.67	125.00	< .001	25.64	15.63	41.67	< .001	0.83	0.60	1.14	0.25
Preterm	0.85	0.58	1.24	0.39	1.36	0.85	2.18	0.20	1.46	0.62	3.44	0.38	2.36	1.56	3.57	< .001
*Complications*																
During pregnancy	0.32	0.18	0.581	< .001	0.99	0.63	1.59	0.99	1.14	0.33	3.88	0.84	0.67	0.37	1.25	0.210
At delivery	1.58	0.88	2.84	0.13	1.19	0.61	2.32	0.61	0.09	0.01	0.85	0.04	32.12	15.98	64.56	< .001
Neonatal	1.12	0.71	1.77	0.62	1.69	1.01	2.84	0.05	0.59	0.22	1.66	0.32	4.85	3.12	7.54	< .001
*Ultrasounds*																
None (ref)	1.0				1.0				1.0				1.0			
1	0.36	0.28	0.48	< .001	1.21	0.89	1.65	0.22	1.24	0.79	1.91	0.34	1.58	1.03	2.42	0.04
≥ 2	0.45	0.33	0.61	< .001	1.35	0.95	1.91	0.09	0.05	0.02	0.09	< .001	1.61	1.01	2.58	0.05
*Tests Done*																
CBC/FBS	0.79	0.52	1.22	0.295	0.29	0.18	0.49	< .001	0.18	0.08	0.42	< .001	0.446	0.252	0.79	0.01
Urinalysis	0.59	0.47	0.77	< .001	0.78	0.59	1.02	0.07	1.25	0.75	2.09	0.39	0.79	0.56	1.12	0.18
Infectious	0.55	0.22	1.35	0.19	0.84	0.26	2.67	0.76	0.62	0.17	2.24	0.47	**	**	**	**

**Due to small cell size for “No” values were not computed

## Discussion

This study highlighted main maternal health outcomes among a large sample of disadvantaged pregnant women, who are refugees (Syrians and Palestinians) or from the host community (Lebanese), seeking ANC services at PHCs in Lebanon.

### Suboptimal ANC visits

Our findings showed that disadvantaged Lebanese and Syrian women, particularly those located in Bekaa, completed a fewer than recommended number of ANC visits throughout their pregnancy, with a significant difference compared to Palestinian pregnant women or those located in other regions of Lebanon. This may be explained by the fact that the Bekaa region hosts the highest number of refugees, namely Syrian refugees, in Lebanon [46]. The Bekaa region is also characterized by an overall inadequate access to health services even prior to the influx of Syrian refugees, due to fragmented infrastructure and lack of essential resources [47]. In fact, Benage et al. reported that Syrian refugees in the Bekaa governorate accessed ANC services less than refugees located in other regions of the country [24]. Many factors and obstacles may have also played a role in leading to this suboptimal number of ANC visits in Bekaa, including the difficulties in transportation that pregnant women may face in reaching the healthcare facilities, and the shortage of female healthcare professionals which is a context- and culture- specific factor [29].

Regarding the age of the pregnant women, we noticed that as age increases, number of ANC visits decreases. This association is consistent a study in Ethiopia that showed that older pregnant women in rural areas were less prone to adequately utilize antenatal care compared to younger pregnant women[48].. Similarly, our analysis revealed that more gravidae and parities were recognized in women with < 4 ANC visits (0.4 and 0.3, respectively *p* < 0.001). A potential explanation is that women with high parity may feel more experienced and self-dependent, they may erroneously assume that they do not need ANC visit. This is disconcerting since those women are at higher risk of complication during pregnancy and delivery [49–51]. Consequently, further investments should be made in educating women, particularly those in fragile settings, about the importance of ANC through the provision of necessary educational information. Such interventions may subsequently correct any existing misinformation that the women may have had about ANC and increase the uptake of ANC services among them. One example of these interventions is the implementation of the Mobile University of Health (MUH)-Women’s Health capacity building initiative by the Global Heath Institute (GHI) Academy, where disadvantaged women from the Lebanese community and Syrian refugees were trained on different health topics -including women’s health- to address health concerns of the surrounding community members (family members, neighbors or friends)[52]. These women were referred to as Community Health Workers (CHWs) applying their knowledge and skills gained from the MUH certificate to spread awareness about women’s health and improve access to healthcare services [52].

### Type of delivery/delivery outcome

A high rate of c-section deliveries (40%) was documented in our study. This figure is consistent with global trends, since despite the fact that such surgical interventions may increase the risk of health issues to the pregnant women and the babies, c-section rates have increased globally from around 7% in 1990 to 21% nowadays, and are expected to reach 29% on 2030 [53]. The utilization of c-section in the absence of medical indication has no benefits and may pose potential harm and inefficiencies in resource allocation. The overuse of procedure-section deliveries could result in unwarranted depletion of human and financial resources, which could have been otherwise allocated more effectively towards achieving better health outcomes [54–56]. Therefore, enhancing the appropriateness of c-section utilization has become a critical global public health issue and a challenge that needs to be addressed [57, 58]. In our study, a higher rate of c-section deliveries was observed in the capital Beirut, this disparity could be explained by socioeconomic, demographic, and healthcare factors including higher income and accessibility to healthcare services in Urban areas [59–61]. A decreased rate of c-section was observed among the Syrian refugees as compared to other nationalities. Syrian refugees receive delivery services through the support of subsidies from the UNHCR [62]. In order for them to undergo c-section, an approval ensuring the presence of a definite medical indication for this procedure is required from a third-party administrator (TPA) [62]. These TPAs bear the responsibility of ensuring the presence of a clear medical necessity for a c-section before granting approval. Consequently, in cases where there is no such medical requirement for a c-section, TPAs withhold their approval, leading to reduced c-section rates among the refugee community. Having the right checks and balances through the enforcement of an approval process to ensure the evidence-based utilization of c-section deliveries, appears to have made a significant difference in the utilization rate. This would enhance the safety of mothers and newborns while keeping the cost of maternal care lower to the funding parties. The authors recommend a more systematic investigation to gain a more comprehensive understanding of the variation in cesarean section rates in Lebanon.

In order to overcome this increase in c-section rates and its consequences, the World Health Organization (WHO) recommends implementing educational programs to help women take an active role in planning their birth, conducting regular audits of c-section practices, using clinical guidelines for interventions and requiring more than one medical opinion to perform a c-section whenever feasible [53]. It is imperative to provide healthcare providers with adequate education to prevent the potential misuse, overuse, or abuse of c-section procedures [63]. Additionally, to accurately track the utilization of resources, a robust monitoring and evaluation system needs to be established. In this regard, funding agencies could collaborate with local partners to institutionalize enhanced controls and audits of the mode of delivery to ensure the optimal allocation of resources. These recommendations are of great importance to the Lebanese context specifically, given the high rates of c-section and the overall low awareness about its implications particularly among disadvantaged populations [64, 65].

As for abortion, our findings underline a significant association between the geographical distribution of the PHCs and the incidence of abortion. Lower rates of abortion were documented in Bekaa, Mount Lebanon, and South Lebanon as compared to Beirut. Given that Beirut is the capital of Lebanon, it’s known for its urbanization and diverse population. It serves as the country's economic, cultural, and political center. Capital cities typically have higher abortion rates due to many factors, including easier access to healthcare facilities and services, such as the abortion services [66]. Secondly, capital cities frequently have greater cultural and social diversity than other areas, which might foster an environment that is more accepting and tolerant for those who might be looking for abortion services. [66]. Another factor that may be leading to higher rates of abortion is the long-term exposure to air pollution [67–69], especially that Lebanon is examining an increase in pollution incidents, mainly in urban neighborhoods such as Beirut [70–73]. Higher rates of abortion were also observed in our study in pregnant women with history of abortion, preterm delivery, and c-section. In line with our findings, a Norwegian study by Magnus et al. showed that history of abortion, preterm delivery and c-section were also associated with an increased risk of current abortion [74]. One possible explanation for the association of the history of c-section and the incidence of abortion is the breech presentation for c-section delivery in the previous pregnancy [75].

### Onset of delivery

The findings of this study underscored that around 6% of the pregnant women had preterm deliveries. History of preterm delivery was shown to be a major contributor to having a preterm delivery in the current pregnancy (OR = 2.36), which aligns with the findings of a study conducted by Yang et al. that showed that prior preterm delivery is strongly associated with the risk of subsequent preterm delivery [76]. This correlation highlights the need for individualized ANC with defined guidelines and recommendations for pregnant women with history of preterm delivery to prevent subsequent preterm deliveries, if possible [77].

Our study also showed a significant association between preterm delivery and undergoing current c-section (OR = 2.85 *p* < 0.001). C-section, which accounts for 19% of preterm deliveries in our findings, is one of the preferred interventions for pregnancy termination in medically indicated preterm delivery [78]. Complications during pregnancy were identified as one of the medical indications for preterm delivery, and our findings showed that complications at delivery increased the likelihood of preterm delivery (OR = 32.12 *p* < 0.001) [79]. Although half of the pregnant women in our study did not undergo ultrasounds screening, those who had one or two or more ultrasounds were found to have higher odds of preterm delivery. Ultrasound assessment is a widely used diagnostic tool that can provide valuable information about the health of a developing fetus, including the detection of preterm delivery [80, 81]. By providing accurate information about fetal gestational age, cervical length, fetal growth, and well-being, ultrasound can help identify women who are at risk for preterm delivery and allow clinicians to provide appropriate care to reduce the risk of complications for both the mother and the baby [81]. Encouraging women to do the recommended ultrasounds may be challenging, yet it is crucial in order to enhance the health of the pregnant woman and the baby. This can be achieved by providing education to the pregnant woman, making ultrasound screening accessible, providing reminders and follow-ups, and involving the healthcare provider in the encouragement process. In our study, CBC and FBS blood testing decreased the odds of preterm delivery by half. Blood testing during pregnancy can help identify certain conditions that can increase the risk of preterm delivery such as anemia [82], gestational diabetes [83] among many other complications during pregnancy. By detecting and managing these conditions early, healthcare providers can take steps to reduce the risk of preterm delivery and its associated complications.

In light of these findings, this study highlights the need for low-cost contextualized interventions focused on improving maternal health and the uptake of ANC services. Such interventions should be co-designed with the target population to ensure human-centered solutions that take into consideration contextual and ethical factors including cost, vulnerability, gender, and power dynamics among others, while adapting the intervention to the specific population needs within a suitable financial framework.

### Limitations

While our study provides a comprehensive overview of maternal health outcomes among pregnant women residing in a fragile setting, several limitations were encountered when conducting this study. One main constraint was the relatively lower-than-usual number of pregnant women who visited the PHCs during their first trimester throughout our period of interest for chart review. This was mainly because the timeframe for the study coincided with the COVID-19 pandemic and lockdown restrictions in Lebanon. Consequently, most of women only visited the centers for the first time towards the end of their third trimester or just before their due date, with the main objective being to obtain a referral to a public or private hospital for delivery (in the case of UNRWA) or to obtain a delivery package comprising essential items for themselves and their newborns (in the case of MOPH). In addition to the COVID-19 pandemic, a number of pregnant women opted to skip their antenatal and/or postnatal appointments due to the challenging economic situation in Lebanon. As a result, they missed some of the necessary tests and scans during their pregnancy period. Moreover, the availability of tests offered at MOPH centers is dependent on external funds. Therefore, if funding is terminated or if insufficient funds are available to cover the full range of tests for each woman, many tests may not be performed at these centers until additional funding is secured. As a result, some patients may experience delays or gaps in their medical care, and may need to seek testing or imaging services elsewhere.

Another limitation of our study was the missing data in the records. In 2019, the MOPH PHCs implemented the Primary Healthcare Network Information and Communication System (PHENICS) electronic health record system, but some centers were unable to transfer all of the historical data from before that time, resulting in lost or incomplete records. Moreover, certain labs and scans were not available at some MOPH and UNRWA centers, leading to pregnant women referral to other facilities for testing and not reporting results back to the PHCs. Failure to provide the PHC with test results by the pregnant women meant that these results were not recorded in the women's medical charts. Furthermore, missing data was also caused by women skipping visits or discontinuing care after delivery, which resulted in incomplete records of antenatal and postnatal complications, delivery details, and gestational age. Some women sought care from private OBGYN clinics, which meant that their records did not always provide a complete picture of their medical history, particularly when test results were not reported to the PHC. Additionally, certain important variables, including previous surgeries, smoking and alcohol consumption, and breastfeeding practices, were not routinely recorded during screening by midwives or nurses in the PHCs.

## Conclusion

In conclusion, this study highlighted three main maternal health outcomes (number of ANC visits, delivery type/outcomes, and delivery onset) among disadvantaged Lebanese, Syrian refugee, and Palestinian refugee pregnant women. The study found that the region of the PHC, the nationality, and the age of the pregnant women were associated with all three maternal health outcomes. Additionally, pregnant women with a history of abortion, c-section delivery, and preterm delivery were more likely to develop the same maternal outcomes with suboptimal ANC visits in their current pregnancy. The study further emphasized the importance of implementing low-cost contextualized interventions that aim to optimize access to ANC visits and enhance the uptake of ANC services among pregnant women, particularly those residing in fragile settings.

## Data Availability

All data generated or analysed during the current study are available from the corresponding author on reasonable request.

## Abbreviations

UN: United Nations
SDGs: Sustainable development goals
NGOs: Non-governmental organizations (NGOs)
PHC: Primary healthcare center
MOPH: Ministry of public health
UNHCR: United Nations high commissioner for refugees
UNRWA: United Nations relief and works agency
ANC: Antenatal care
PNC: Postnatal care
MUH: Mobile University of health
GHI: Global health institute
CHWs: Community health workers
TPA: Third-party administrator
WHO: World Health Organization
PHENICS: Primary healthcare network information and communication system
